# Endoscopic ultrasound-guided drainage of bilomas in difficult-to-puncture locations using a sheath-assisted puncture technique

**DOI:** 10.1055/a-2418-3400

**Published:** 2024-10-14

**Authors:** Kazuki Hama, Yukitoshi Matsunami, Takayoshi Tsuchiya, Reina Tanaka, Ryosuke Tonozuka, Shuntaro Mukai, Takao Itoi

**Affiliations:** 1 13112 Department of Gastroenterology and Hepatology, Tokyo Medical University, Shinjuku-ku, Japan

Biloma is a complication of transcatheter arterial chemoembolization (TACE) for hepatocellular carcinoma (HCC) 1 . Despite being the first-line treatment for infected bilomas, percutaneous drainage can affect daily life and pose self-extraction risks, particularly in older patients 2 . Endoscopic ultrasound (EUS)-guided drainage offers a viable alternative for internal biliary drainage; however, it can be challenging for the right liver lobe because of its long distance from the gastrointestinal (GI) tract 3 4 5 . Here, we describe successful EUS-guided drainage of an infected biloma distant from the GI tract using a sheath-assisted puncture technique ( Fig. 1 , Video 1 ).

**Fig. 1:**
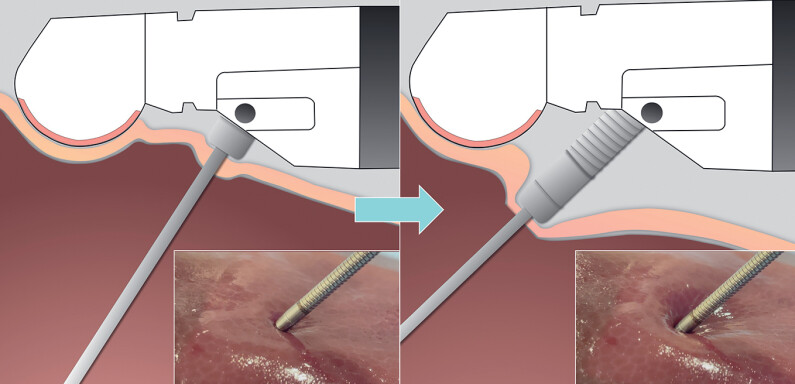
Sheath-assisted puncture technique allows the needle tip to reach deeper puncture targets by pushing the sheath of a 19G needle. The 2.6-mm sheath prevented damage to the gastric mucosa and liver parenchyma.

Sheath-assisted puncture technique allows the needle tip to reach deeper puncture targets by pushing the sheath of a 19G needle.Video 1

An 85-year-old man who developed an infected biloma in the posterior liver segment after TACE for HCC ( Fig. 2 **a** ) opted for endoscopic transpapillary drainage because of advanced age and high risk of self-extraction. However, uncontrollable infections necessitated EUS-guided transduodenal drainage with a nasal drainage tube. Biloma recurrence in segment 7 induced an initial reintervention attempt utilizing a guidewire along the tube ( Fig. 2 **b** ). Difficulty in passing the guidewire into the biloma cavity prompted additional EUS-guided transduodenal drainage. A convex EUS scope (GIF-UCT260; Olympus, Tokyo, Japan) visualized the deeper residual biloma, which required puncturing. However, the 19G needle (EZ shot 3 plus; Olympus) did not reach the target, even at its maximum extent ( Fig. 3 **a** ). A deep puncture was made to reach the target by pushing the sheath ( Fig. 3 **b** ). After reconfirming the needle tip in the biloma using contrast, a 0.025-inch guidewire (VisiGlide II; Olympus) was placed, followed by insertion of a 5-Fr nasal drainage tube ( Fig. 2 **c, d** ).

**Fig. 2:**
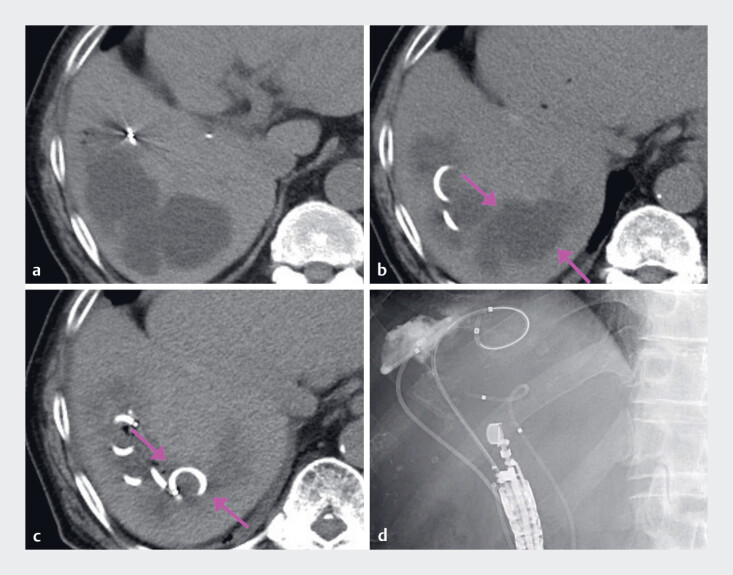
**a** Computed tomography (CT) revealing the biloma in segment 7 (S7) after transcatheter arterial chemoembolization for hepatocellular carcinoma. **b** Endoscopic ultrasound drainage of the S7 was performed, but the infection was uncontrollable (arrow). **c** Postoperative CT showing that the biloma had shrunk and infection was well controlled (arrow). **d** X-ray confirming that the nasal drainage tube was well positioned.

**Fig. 3:**
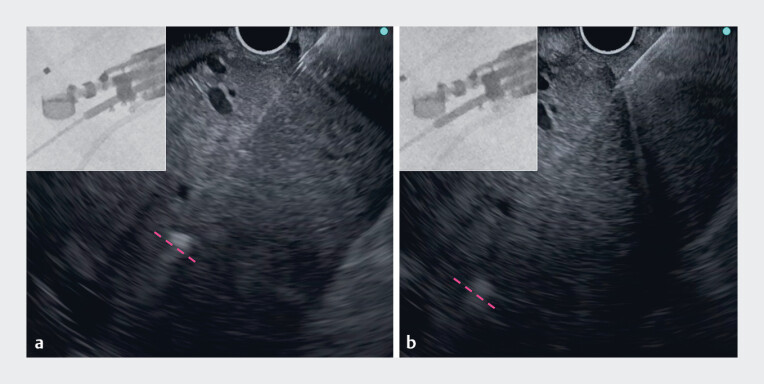
**a** A 19G needle was used to puncture the deeper infected biloma, but the needle did not reach the biloma even when it was pushed out to its maximum extent (dotted line shows the needle tip). **b** The needle tip reached the biloma using a sheath-assisted puncture technique.

Post-procedural computed tomography confirmed biloma shrinkage and well-controlled infection. The patient was discharged on postprocedural day 10 with no adverse events. The 19G needle has a 2.6-mm sheath, which allows safe sheath-assisted puncture without damaging the gastric mucosa and liver parenchyma.

Endoscopy_UCTN_Code_TTT_1AS_2AH

## Notes

Endoscopy E-Videos https://eref.thieme.de/e-videos
*E-Videos* is an open access online section of the journal *Endoscopy* , reporting on interesting cases and new techniques in gastroenterological endoscopy. All papers include a high-quality video and are published with a Creative Commons CC-BY license. Endoscopy E-Videos qualify for HINARI discounts and waivers and eligibility is automatically checked during the submission process. We grant 100% waivers to articles whose corresponding authors are based in Group A countries and 50% waivers to those who are based in Group B countries as classified by Research4Life (see: https://www.research4life.org/access/eligibility/ ). This section has its own submission website at https://mc.manuscriptcentral.com/e-videos .
